# Supplementary material for: Instruments for Assessing Nursing Care Quality: A Scoping Review
Source: Nurs Rep. 2025 Sep 19;15(9):342. doi: 10.3390/nursrep15090342 (PMC12472527; doi:10.3390/nursrep15090342)
Supplement: Supplementary file 1 [file nursrep-15-00342-s001.zip › nursrep-3829643-supplementary.pdf]

Supplementary Table S1 – Search Strategy

	Natural Language	MeSH	CINAHL
**Population (P)**	Tool* OR instrument* OR scale* OR questionnaire*	----------------------	Scales OR Questionnaires
**Concept (C)**	Quality	"Quality of Health Care" OR "Quality Assurance, Health Care"	"Quality of Nursing Care" OR "Quality of Health Care" OR "Quality Assurance" OR "Quality Assessment"
**Context (C)**	"nursing care"	"nursing care"	"nursing care"

✓ ***CINAHL Complete* (*EBSCO Host*)**

Date: 10 de fevereiro 2025

Search	Query	Findings
S1	TI (Tool* OR instrument* OR scale* OR questionnaire*) OR MH (Scales OR Questionnaires)	941,998
S2	TI Quality OR MH ("Quality of Nursing Care" OR "Quality of Health Care" OR "Quality Assurance" OR "Quality Assessment")	247,278
S3	TI "nursing care" OR MH "nursing care"	39,474
S4 (S1 AND S2 AND 3)	(TI (Tool* OR instrument* OR scale* OR questionnaire*) OR MH (Scales OR Questionnaires)) AND (TI Quality OR MH ("Quality of Nursing Care" OR "Quality of Health Care" OR "Quality Assurance" OR "Quality Assessment")) AND (TI "nursing care" OR MH "nursing care")	876

✓ **PubMed**

Date: 10 de fevereiro 2025

Search	Query	Findings
S1	Tool*[Title] OR instrument*[Title] OR scale*[Title] OR questionnaire*[Title]	350,678
S2	((Quality[Title]) OR ("Quality of Health Care"[Mesh:NoExp])) OR ("Quality Assurance, Health Care"[Mesh:NoExp])	413,305
S3	("nursing care"[Title]) OR ("nursing care"[MeSH Terms])	151,630
S4 (S1 AND S2 AND 3)	((Tool*[Title] OR instrument*[Title] OR scale*[Title] OR questionnaire*[Title]) AND (((Quality[Title]) OR ("Quality of Health Care"[Mesh:NoExp])) OR ("Quality Assurance, Health Care"[Mesh:NoExp]))) AND (("nursing care"[Title]) OR ("nursing care"[MeSH Terms]))	241

- ✓ ***Scopus***

Date: 10 de fevereiro 2025

Search	Query	Findings
S1	(TITLE (tool* OR instrument* OR scale* OR questionnaire*) AND TITLE (quality) AND TITLE ("nursing care"))	68

- ✓ ***Web of Science***

Date: 10 de fevereiro 2025

Search	Query	Findings
S1	TI=(Tool* OR instrument* OR scale* OR questionnaire*) AND TI=(Quality) AND TI=(“nursing care”)	43

- ✓ ***ProQuest Dissertations & Theses Citation Index (Web of Science)***

Date: 10 de fevereiro 2025

Search	Query	Findings
S1	TI=(Tool* OR instrument* OR scale* OR questionnaire*) AND TI=(Quality) AND TI=(“nursing care”)	5
